# Interventions of Advanced Lung Cancer Patient Receiving Chemotherapy by Computed Tomography Image Information Data Analysis-Based Soothing Care Plans

**DOI:** 10.1155/2022/3585567

**Published:** 2022-06-09

**Authors:** Juan Wang, Shuangping Lu, Qundan Zhang

**Affiliations:** Department of Respiratory Medicine, First People's Hospital of Fuyang District, Hangzhou, 311400 Zhejiang, China

## Abstract

The objective of this study was to investigate the intervention effect of computed tomography (CT) image information data on patients with advanced lung cancer treated with chemotherapy under palliative care program. The research subjects were 60 patients with advanced lung cancer who received palliative care in our hospital from January 1, 2019, to January 1, 2021. All patients were grouped according to the evaluation criteria of solid tumor efficacy, including 28 patients in the remission group and 32 patients in the nonremission group. Texture analysis was performed on the CT images of the two groups of patients. The gray-scale cooccurrence matrix parameters, the maximum diameter of the lesion, and the CT value of the CT images of the two groups of patients before and after palliative care were compared. The results showed that after the palliative care, the combined mean, combined energy, and inverse moment of the three gray cooccurrence matrix parameters of the two groups of patients were decreased, and the combined entropy and contrast were increased. The absolute value of the gray-scale cooccurrence matrix Δ parameter of the patients in the remission group was greater than that in the nonremission group. The Δ combined entropy, Δ contrast, and Δ correlation of the two groups of patients were significantly different, and the difference in Δ contrast was the largest. It suggested that the gray-scale cooccurrence matrix parameter can evaluate the effect of soothing care, and the contrast was the best evaluation parameter. The maximum diameter of the lesions in the remission group before and after palliative care was reduced by 1.23 cm, and the degree of reduction was significantly better. The CT value was reduced by 6.22 HU, and the degree of reduction was significantly higher than that in the nonremission group. There was a significant difference in the data between the two groups (*P* < 0.05). Therefore, the CT image information data had a better evaluation effect on patients with advanced lung cancer under the palliative care program and can be applied to the clinical evaluation of the palliative care effect, which had good clinical value.

## 1. Introduction

Lung cancer patients are generally found late, and more than 50% of patients have already reached the advanced stage of lung cancer when they are found [1]. Patients with advanced cancer will suffer great pain, both physically and psychologically. Targeted nursing measures are extremely necessary, which can relieve the pain of patients and improve the acceptance and satisfaction of patients. It is found that patients with lung cancer basically need chemotherapy treatment. Chemotherapy is a treatment method that seriously damages the body and will bring pain to patients in both physical and psychological aspects [2]. Lung cancer patients often suffer from dyspnea and chest tightness due to severe impairment of lung function, and even vomiting blood in severe cases, which will bring pain and panic to patients and make it difficult for patients to live a normal life [3]. In recent years, palliative care has gradually been welcomed and recognized in the care and treatment of patients, and it is a new concept of nursing. It advocates respecting the wishes of patients, caring for the psychological needs of patients, providing patients with comfortable and humanistic nursing services, meeting the individual needs of patients, and maximizing pain relief [4]. Palliative care is mainly to maximize the comfort of patients, maintain the dignity of patients, and improve the quality of life of patients.

Computed tomography (CT) and pathological examination are the main methods of tumor examination. The sampling of pathological and biological lesions is invasive, which brings harm to patients, and is prone to puncture complications, only the pathological conditions of the lesion tissue are obtained, and there is no complete lesion information. CT is also required for long-term efficacy monitoring [5]. CT examination is currently a widely used technique, which can play a guiding role in the evaluation of the condition of lung cancer and the evaluation of treatment effectiveness [6]. CT technology can intuitively and comprehensively display the size and changes of the tumor, and doctors can evaluate the treatment and nursing effect of lung cancer patients by observing the images. The disadvantage is that the level and diameter of image measurement are subjectively determined by the doctor, which will be affected by the doctor's judgment, and is highly dependent on the doctor's specialization and technology [7]. There are different changes in the structure of the lesions in each direction of the three-dimensional space. Therefore, the diameter measurement of the planarized single-dimensional and single-layer cannot fully display the detailed changes of the lesions in a three-dimensional and complete manner [8]. The texture analysis of CT images can obtain the parameters related to the microstructure of the lesions. On the basis of conventional imaging, the entire lesions can be analyzed through the parameters, and the spatial heterogeneity of the lesions can be displayed. It is widely used in the medical field and has a high value [9, 10].

In this study, it analyzed the texture analysis of CT images before and after nursing in the remission group patients and nonremission group patients and compared the gray-scale co-occurrence matrix parameters, the maximum diameter of the lesions, and the CT value to evaluate the effect of palliative care. In addition, the CT image information data was applied to the evaluation of nursing effect aims to provide a choice of evaluation methods for the evaluation of the effect of lung cancer patients, showing positive clinical value in the diagnosis and treatment of lung cancer patients.

## 2. Materials and Methods

### 2.1. Research Objects

The subjects included in this study were 60 patients with advanced lung cancer who were admitted to the hospital from January 1, 2019, to January 1, 2021. All patients were grouped according to the evaluation criteria of solid tumor efficacy, including 28 patients in the remission group. The male to female ratio was 16 : 12, with an average age of 67.73 ± 6.38 years old. There were 32 patients in the nonremission group, with a male to female ratio of 18 : 14 and an average age of 67.89 ± 6.33 years old. Patients were treated with chemotherapy and palliative care for 2 months. There was no statistical difference in general data between the two groups (*P* > 0.05). This study was approved by the ethics committee of hospital.

Inclusion criteria were defined as follows: patients who were diagnosed with lung cancer; patients with clinical stage III or IV according to the 8th edition of the Union for International Cancer Control (UICC) TNM staging recommendation for lung cancer, patients treated with chemotherapy, patients whose CT showed nodules or masses with clear borders, patients whose CT data were complete, and patients who signed the informed consents.

Exclusion criteria were given as follows: patients whose images with cavities ≥5 mm, patients whose images with pulmonary vessels of ≥3 mm passing through, patients whose images with calcified hemorrhagic foci, and patients with severe CT image artifacts.

### 2.2. CT Scan and Image Reconstruction

Definition AS Siemens 64-slice CT instrument was used (Siemens, Germany). Before the examination, patients were asked to do respiration training to eliminate their tension and avoid image artifacts caused by patients' poor cooperation. During the examination, patients were assisted to take supine position and raise arms. At the end of breathing, scanning was carried out. The scanning range was from lung apex to lower level of costophrenic angles and thoracic walls as well as axillary fossa. The scanning parameters were set as follows. Tube voltage was 120 kV, collimation width was 128 × 0.625 mm, pitch was 0.993, the rotation time of bulb tube was 0.75 s, scanning view was 400 mm, and acquisition matrix was 512 × 512. The reconstruction parameters were set as follows. Reconstruction thickness was 5 mm, reconstruction interval was 5 mm, window level was 35 HU, and window width was 250 HU. Iteration algorithm was adopted to reconstruct images, and filtering function was on standard mode. The obtained CT images were observed under mediastinum windows. The methods of enhancing scanning were as follows. High-pressure injector was utilized to inject ioversol through elbow veins (320 mgL/mL). Adult dose was 1.5 to 2.0 mL/kg, injection rate was 2.5 to 3.0 mL/s, and delay time was 55 to 90 s.

### 2.3. Data Analysis of CT Image Information

The images were preprocessed by gray-level quantification, the CT images were segmented by Siemens radiomics tools, and the region of interest (ROI) of the tumor tissue was manually delineated. When the sketch was completed, Philips Radiomics Tool was utilized to extract the texture feature parameters of CT images. In addition, computer software was used to calculate GLCM parameters. Cooccurrence matrix consists of the combined probability density *P*(*a*, *b*, *d*, *θ*) among image gray levels and reflects the spatial relevance of the gray levels between two arbitrary points in images. *β* and *r* were defined as direction and interval, respectively, and GLCM was [*P*(*a*, *b*, *r*, *β*)]*L* × *L*. *P*(*a*, *b*, *r*, *β*) referred to the value of the element at the a^th^ row and the b^th^ column in cooccurrence matrix. The value denoted the probability of the emergence of gray level *b* with gray level *a* selected as the starting point and the given spatial distance *r* as well as direction *β*. Besides, GLCM was assigned with *r* = 1 and *β* = 0°.

A total of 6 GLCM parameters were selected, including combined mean expressed by *k*1, combined entropy expressed by *k*2, combined energy expressed by *k*3, contrast ratio expressed by *k*4, inverse difference moment expressed by *k*5, and relevance expressed by *k*6. The calculation methods of GLCM-related parameters were shown in equations (1) to (10) below. Besides, the value of Δ parameter was calculated, as equation (11) demonstrated below.

(1) $$\begin{array}{c} k1=\underset{\alpha }{\sum }\underset{b}{\sum }P\left(a,b\right)a, \end{array}$$

(2) $$\begin{array}{c} k2=-\underset{a}{\sum }\underset{b}{\sum }P\left(a,b\right)\log_{2}P\left(a,b\right), \end{array}$$

(3) $$\begin{array}{c} k3=\underset{a}{\sum }\underset{b}{\sum }{\left(P\left(a,b\right)\right)}^{2}, \end{array}$$

(4) $$\begin{array}{c} k4=\underset{n}{\sum }n^{2}\left\{\underset{a}{\sum }\underset{b}{\sum }P\left(a,b\right)\right\},n=\left|a-b\right|, \end{array}$$

(5) $$\begin{array}{c} k5=\underset{a}{\sum }\underset{b}{\sum }\frac{P\left(a,b\right)}{1+{\left(a-b\right)}^{2}}, \end{array}$$

(6) $$\begin{array}{c} k6=\underset{\alpha }{\sum }\underset{b}{\sum }\frac{abP\left(a,b\right)-w_{1}w_{2}}{\theta_{1}^{2}\theta_{2}^{2}}, \end{array}$$

(7) $$\begin{array}{c} w_{1}=\underset{a}{\sum }a\underset{b}{\sum }P\left(a,b\right), \end{array}$$

(8) $$\begin{array}{c} w_{2}=\underset{a}{\sum }b\underset{b}{\sum }P\left(a,b\right), \end{array}$$

(9) $$\begin{array}{c} \theta 1=\underset{a}{\sum }{\left(a-w_{1}\right)}^{2}\underset{b}{\sum }P\left(a,b\right), \end{array}$$

(10) $$\begin{array}{c} \theta 2=\underset{a}{\sum }{\left(a-w_{2}\right)}^{2}\underset{b}{\sum }P\left(a,b\right), \end{array}$$

(11) $$\begin{array}{c} ∆\text{parameter}=\text{After treatment}-\text{Before treatment}. \end{array}$$

### 2.4. Soothing Care

All patients were performed with chemotherapy and soothing care measures. The specific measures were as follows.

- Soothing nursing groups were set up, and head nurse was selected as group leaders. The group leaders organized regular theoretical and practical training for nursing staff to improve their tumor specialized nursing theoretical knowledge level. Besides, their experience was accumulated, and practical ability as well as nursing effects were enhanced combined with relevant cases
- The difficulty levels of respiration of lung cancer patients were assessed, and corresponding nursing intervention measures were implemented for patients. If patients suffered from anhelation, they could be assisted to take semireclining position, orthopnea position, or other comfortable positions. In addition, patients were instructed to relax upper chests and shoulders to enable abdominal walls and diaphragms to expand. If patients suffered from accelerated breathing, cyanosis, and elevated blood pressure, patients needed to be offered oxygen inhalation in time and bronchiectasis drug treatment following doctors' advice. Respiratory control skill training was carried out for patients. Patients were asked to place left hands on chests and right hands on navels of abdomens. During the inhalation, patients' abdomens needed to be expanded as out as possible with chests being kept still. During the exhalation, abdomens needed to be retracted as much as possible. In the training processes with different actions, the pace of respiration should be kept the same. What is more, respiratory relaxation technique training was implemented for patients. Patients were asked to relax cervical muscles during respiration. Besides, chest muscle motion should be avoided. Lip contraction respiration method was demonstrated for patients. Patients were instructed to make a whistling shape with their lips. Tongue apex was placed at the inner base of underjaw teeth, and tongue body was kept close to the junction of soft and hard palates of maxilla. Inhalation was completed with nostril, and exhalation was finished through mouth with whistling posture
- Patients were provided with comfortable hospitalization environment. Wards needed to be spacious and bright with regular ventilation and continuous air circulation. Humidity level should be kept between 55% and 60% and temperature needed to be kept between 20°C and 22°C. In addition, nursing staff played soothing music for patients to alleviate their anxiety so that they felt relaxed
- Patients were offered mental nursing. Nursing staff should pay attention to patients' mental state in time, communicate with patients actively, understand patients' needs, solve the problems patients were faced with, encourage and soothe patients, relieve patients' negative emotions, and reduce the adverse impacts of mental stress on patients

### 2.5. Observation Indexes

The statistics of the response effects on advanced lung cancer patients after soothing care was conducted, and the evaluation criteria were response evaluation criteria in solid tumors (RECIST). The criteria were divided into four types, including complete disappearance of lesions evaluated as complete response (CR), the reduction of the maximum diameter of target lesions at least by 30% evaluated as partial response (PR), the failure of the reduction of target lesions in reaching PR level or the increase in reaching PD level evaluated as stable disease (SD), and the increase of the maximum diameter of target lesions at least by 20% and the increase of diameter at least by 5 mm evaluated as progressive disease (PD). Patients with PR and CR were assigned to remission group, and those with SD and PD were assigned to unresponsive group.

Before and after the soothing care intervention for patients, data analysis of patients' CT image information was carried out. A total of six GLCM parameters, including combined mean, combined entropy, combined energy, contrast ratio, inverse difference moment, and relevance, were calculated by computer software. Besides, Δparameters before and after the care were calculated.

Six GLCM parameters and Δparameter receiver operating characteristic (ROC) curves were drawn before and after the care. The maximum diameter of CT image lesions of patients and CT values was calculated before and after the care.

### 2.6. Statistical Methods

Statistical product and service solutions (SPSS) 20.0 was utilized for statistics and analysis. ROC curves were drawn to evaluate the intervention effects of CT image information data on advanced lung cancer patients receiving chemotherapy with soothing care. Data were compared by *t* test, and *P* < 0.05 indicated that the differences were significant and showed statistical meaning.

## 3. Results

### 3.1. Statistics of Patients' General Data and Information

**Figure 1:**
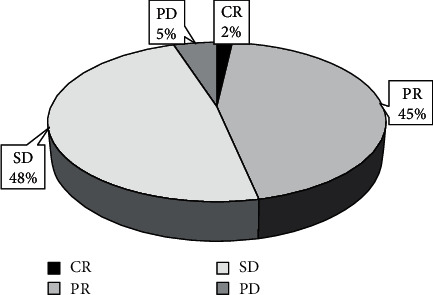
Statistics of patients' general data and information.

Figure 1 showed the results of the assessment of therapeutic effects of soothing care on 60 included advanced lung cancer patients below. According to Figure 1, there was a total of 1 CR patient accounting for 2% of the total number of included patients. There were 27 PR patients, which occupied 45% of the total number of included patients. The proportion of SD patients (29 in total) in all included patients reached 48%. Besides, there were total 3 PD patients with accounting for 5% of the total number of all included patients. Remission group included 28 patients in total with the proportion being 47%. In unresponsive group, there were total 32 patients, accounting for 53% in the total number of all included patients.

### 3.2. Comparison of CT Image GLCM Parameters of Patients in Two Groups before and after Care

**Figure 2:**
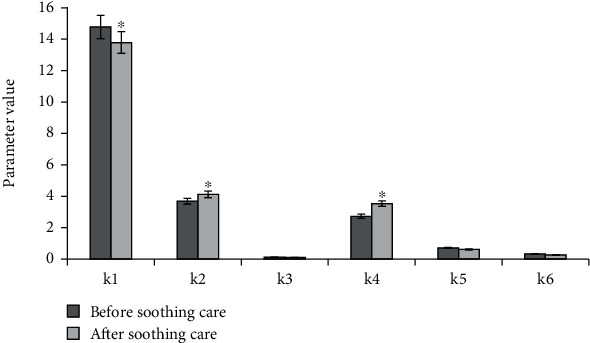
Comparison of GLCM parameters of CT images before and after palliative care in remission group patients. ∗Compared with before palliative care, *P* < 0.05.

**Figure 3:**
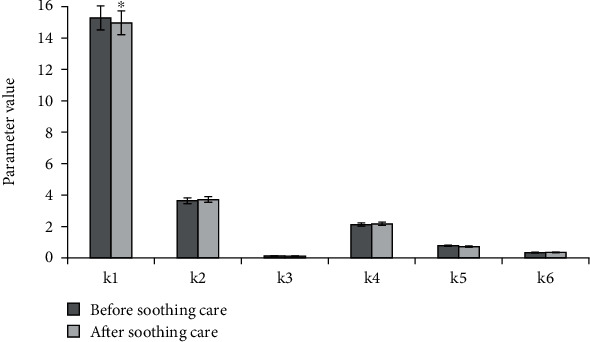
Comparison of GLCM parameters of CT images before and after palliative care in nonremission group patients. ∗Compared with before palliative care, *P* < 0.05.

Figure 2 demonstrated the comparison of CT image GLCM parameters of patients in remission group before and after care, and Figure 3 displayed the comparison of CT image GLCM parameters of patients in unresponsive group before and after care. According to Figures 2 and 3, combined means, combined energy, and inverse difference moments of patients in two groups were all reduced after soothing care. In contrast, combined entropy and contrast ratio of patients in two groups were both increased. In remission group, the relevance was decreased. The relevance of unresponsive group was slightly increased.

### 3.3. Comparison of GLCM ΔParameters of Patients in Two Groups

**Figure 4:**
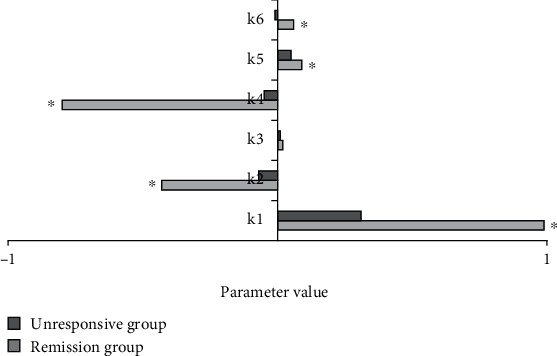
Comparison of GLCM parameters between two groups of patients. ∗Compared with nonremission group, *P* < 0.05.

Figure 4 displayed the comparison of GLCM Δparameters of patients in two groups below. According to Figure 4, the absolute value of GLCM Δparameters of patients in remission group was greater than that of patients in unresponsive group. Besides, the differences in Δparameters of the combined entropy, contrast ratio, and relevance of patients in two groups were significant. ΔContrast ratio of remission group was 16 times higher than that of unresponsive group, while Δcombined entropy and Δrelevance were both 6 times higher than those of unresponsive group.

### 3.4. Comparison of GLCM Parameter ROC Curves before and after Care

**Figure 5:**
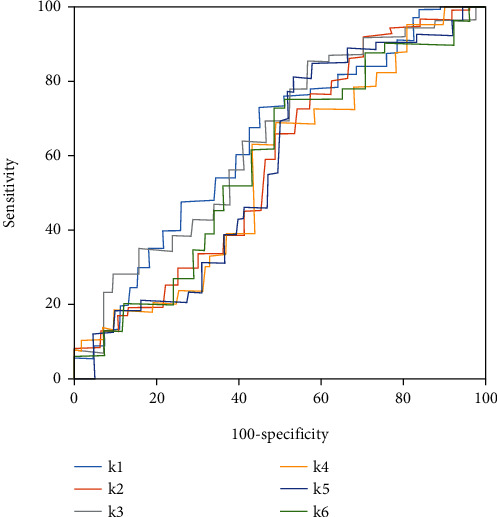
ROC curves of assessment of nursing effects by GLCM parameters of patients before care.

**Figure 6:**
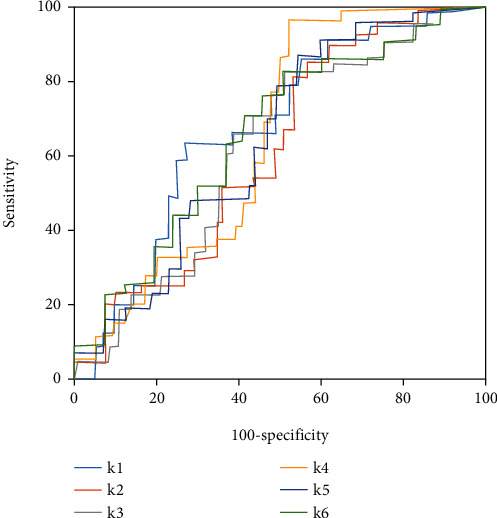
ROC curves of assessment of nursing effects by GLCM parameters of patients after care.

Figure 5 shows GLCM parameter ROC curves before care, and Figure 6 presented GLCM parameter ROC curves of patients after soothing care. According to Figures 5 and 6, the diagnostic effects of each parameter were poor before soothing care. After soothing care, the diagnostic effects of each GLCM parameter were excellent. Among different parameters, combined mean and contrast ratio showed better diagnostic effects. The two parameters could better assess the nursing effects of soothing care on lung cancer patients.

### 3.5. ROC Curve Analysis of Assessment of Nursing Effects by GLCM ΔParameters

**Figure 7:**
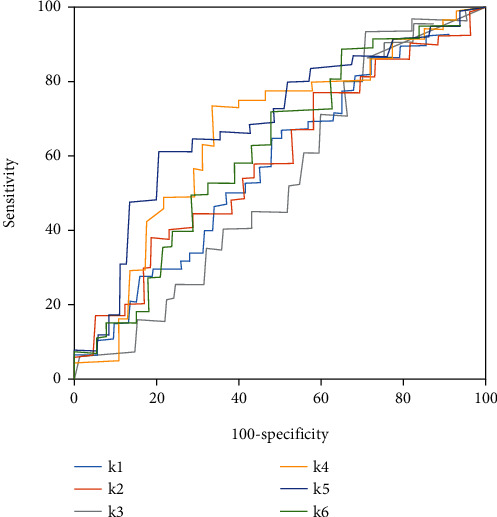
ROC curves of assessment of nursing effects by GLCM Δparameters.

Figure 7 showed ROC curves of the assessment of nursing effects by GLCM Δparameters. According to Figure 7, Δinverse difference moments, Δcontrast ratio, and Δrelevance showed better assessment of soothing care effects. ΔContrast ratio demonstrated the best assessment and could better assess the nursing effects of soothing care on lung cancer patients.

### 3.6. Maximum Diameter of Lesions and CT Values of Patients in Two Groups

**Figure 8:**
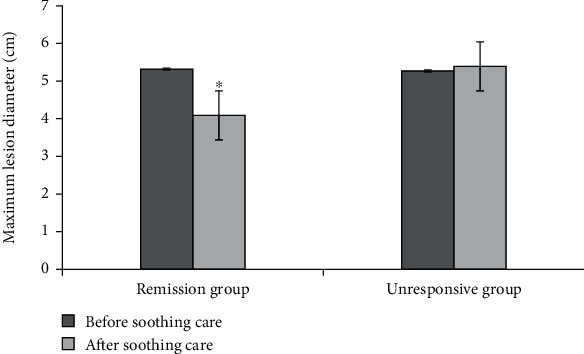
Comparison of the maximum diameter of lesions between the two groups before and after palliative care. ∗Compared with the nonremission group, *P* < 0.05.

**Figure 9:**
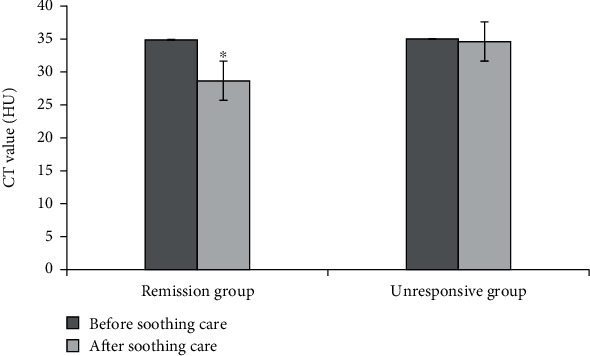
Comparison of CT values before and after palliative care between the two groups of patients. ∗Compared with nonremission group, *P* < 0.05.

Figure 8 demonstrated the comparison of the maximum diameters of lesions of patients in two groups before and after soothing care.  Figure 9 showed the comparison of CT values of patients in two groups before and after soothing care. According to Figures 8 and 9, the maximum diameters of lesions of patients in remission group before and after soothing care were obviously reduced. After soothing care, the maximum diameter of lesions of patients in unresponsive group was slightly increased. The data of patients in two groups were significantly different (*P* < 0.05). In addition, CT value of patients in remission group was obviously reduced after soothing care. In contrast, CT value of patients in unresponsive group was not decreased obviously after soothing care. The data of patients in two groups were significantly different (*P* < 0.05).

## 4. Discussion

Lung cancer has the clinical characteristics of rapid disease progression, poor prognosis, and high mortality [11]. The early symptoms of patients are cough, chest pain, etc., and late stage patients will have symptoms of tumor metastasis and dyspnea, which seriously affects the normal life of patients [12]. When the patient's respiratory function is seriously obstructed, there will be a sense of impending death, which makes the patient feel pessimistic and painful and produces a sense of fear [13]. Soothing and targeted care for patients with lung cancer can improve their pessimism, make them feel warm, and stabilize their psychological state [14]. Palliative care is mainly through helping patients take skills and measures to relieve the pain of the disease, providing psychological comfort to patients, relieving patient pain, oxygen therapy for patients with dyspnea, and discharge guidance for discharged patients, so that patients can experience caring and respect, improving the patient comfort and enabling patients to actively receive care and treatment, thereby improving treatment outcomes [15, 16].

CT examination is a widely used examination method for lung cancer patients. It has the advantages of fast scanning, clear image, simple operation, high repeatability, safety, low price, and high image resolution [17–19]. CT images can clearly reflect the size, shape, and contour of the tumor in the lung field and can also show the internal situation of the tumor and its relationship with surrounding organs [20]. CT scan images can show the lesions in an all-round way, indicating the location, size, density, edge status, etc. of the lesions, which has good clinical application value [21]. Trajanovski et al. (2021) [22] used deep learning to assess cancer risk in CT lung screening and found that low-dose CT screening of the chest could significantly reduce patient mortality, and CT examination under the deep learning model had a positive effect on lung cancer risk assessment. Chen et al. (2021) [23] found that the CT plain scan texture analysis technology can better distinguish benign and malignant pulmonary nodules, which has important value in the risk assessment of lung cancer. This research mainly uses the gray-scale cooccurrence matrix parameters such as combined mean, combined entropy, combined energy, contrast, inverse moment, correlation, and other gray-scale cooccurrence matrix to analyze image texture. The patient's maximum diameter and CT value of the lesion were calculated to evaluate the patient's palliative care effect. The results showed that after palliative care, the combined mean, combined energy, and inverse moment of both groups decreased, combined entropy and contrast increased, and the correlation of remission group decreased. The absolute value of the gray-scale cooccurrence matrix Δ parameter of the remission group was larger than that of the nonremission group, and the Δ contrast difference of the remission group was the largest. It suggested that the gray-scale cooccurrence matrix parameter can evaluate the effect of palliative care, and the contrast evaluation effect was better. In the remission group, the maximum diameter of the lesion was 5.32 cm before palliative care and 4.09 cm after nursing, the reduction degree was significantly better, and there was a significant difference in the data between the two groups (*P* < 0.05). The CT value of the patients in the remission group before palliative care was 34.89 HU, and the CT value after palliative care was 28.67 HU, which was significantly lower than that in the nonremission group. The maximum diameter of the lesion and CT value can better evaluate the effect of palliative care in patients with lung cancer and can be used for auxiliary diagnosis of the treatment effect of lung cancer in clinical practice.

## 5. Conclusion

In this study, the CT image information was adopted to evaluate the effect of lung cancer patients under palliative care. The results showed that CT image information had a good evaluation value for the effect of palliative care, and the gray-scale cooccurrence matrix parameter, the maximum diameter of the lesion, and the CT value can all evaluate the effect of palliative care in patients with lung cancer. Among them, the contrast evaluation had a better effect and can be practiced and applied in clinical practice. The research results showed a positive guiding significance for the diagnosis of lung cancer and the evaluation of the treatment effect. The shortcomings of this work were that the study was carried out in only one hospital, the sample is small, and further research and verification were needed.

## Data Availability

The data used to support the findings of this study are available from the corresponding author upon request.
